# Health literacy, perceived stress, and health-related quality of life in Finnish adults: differences by chronic disease burden

**DOI:** 10.1093/heapro/daag157

**Published:** 2026-10-21

**Authors:** Jing Zhou, Hanna Rekola, Tommi Tolmunen, Timo Lakka, Ismo Linnosmaa, Marjorita Sormunen, Juho Strömmer, Tomi Mäki-Opas

**Affiliations:** School of Law, Shanghai Lixin University of Accounting and Finance, 995 Shangchuan Road, Pudong New District, Shanghai 201209, China; Department of Social Sciences, University of Eastern Finland, P.O. Box 1627, FI-70211 Kuopio, Finland; Department of Social Sciences, University of Eastern Finland, P.O. Box 1627, FI-70211 Kuopio, Finland; Social, Wellbeing, and Rescue Research Centre, Wellbeing Services County of North Savo, P.O. Box 1711, FI-70211 Kuopio, Finland; Institute of Clinical Medicine, School of Medicine, University of Eastern Finland, P.O. Box 100, FI-70029 Kuopio, Finland; Institute of Biomedicine, School of Medicine, University of Eastern Finland, P.O. Box 1627, FI-70211 Kuopio, Finland; Department of Clinical Physiology and Nuclear Medicine, Kuopio University Hospital, P.O. Box 1711, FI-70211 Kuopio, Finland; Kuopio Research Institute of Exercise Medicine, Yliopistonrinne 3, FI-70210 Kuopio, Finland; Department of Health and Social Management, Faculty of Social Sciences and Business Studies, University of Eastern Finland, P.O. Box 1627, FI-70211 Kuopio, Finland; Department of Nursing Science, University of Turku, Medisiina B, FI-20014 Turun yliopisto, Finland; Institute of Public Health and Clinical Nutrition, University of Eastern Finland, P.O. Box 1627, FI-70211 Kuopio, Finland; Wellpro Impact Solutions Oy, Kauppakatu 39, FI-40100 Jyväskylä, Finland; Department of Social Sciences, University of Eastern Finland, P.O. Box 1627, FI-70211 Kuopio, Finland; Social, Wellbeing, and Rescue Research Centre, Wellbeing Services County of North Savo, P.O. Box 1711, FI-70211 Kuopio, Finland

**Keywords:** health literacy, health-related quality of life, perceived stress, chronic disease, multimorbidity, path analysis

## Abstract

Health literacy is associated with health-related quality of life (HRQoL), but the role of perceived stress and possible differences by chronic disease burden remain unclear. We used data from a cross-sectional 2024 quota-based online panel survey of 4003 Finnish adults. The path models included 3716 participants with complete data and used post-stratification weights based on age group and gender. Health literacy was assessed with the HLS-Q12, perceived stress with the PSS-10, and HRQoL with the EuroQol five-dimension, five-level instrument (EQ-5D-5L). Path analysis estimated direct, indirect, and total associations, and multigroup models compared the main associations across three chronic disease-burden groups, adjusting for age, gender, education, employment status, and financial strain. Higher health literacy was associated with lower perceived stress, and higher perceived stress with poorer HRQoL. The indirect association through perceived stress represented approximately four-fifths of the total association based on unstandardized coefficients. The negative perceived stress-HRQoL association became progressively stronger across disease-burden groups on the unstandardized metric and was approximately twice as large among adults with multiple chronic diseases as among those with no chronic disease. The health literacy-stress association was similar across groups. These findings identify a stress-related pattern within the health literacy-HRQoL association and suggest that the association between stress and HRQoL may be particularly important among adults with multiple chronic diseases. Integrated health promotion approaches combining accessible communication, navigation support, stress-sensitive care, and efforts to reduce treatment burden warrant prospective evaluation.

Contribution to Health Promotion

- In a national survey of Finnish adults, people with higher health literacy reported lower stress and better health-related quality of life.
- The indirect association through perceived stress represented a large share of this relationship, but the cross-sectional design cannot establish cause and effect.
- The relationship between stress and quality of life differed by chronic disease burden and was strongest among adults with multiple chronic diseases.
- Health promotion responses could combine accessible information, help with navigating services, and stress-sensitive support. Such approaches require prospective evaluation.

## Background

Health literacy refers to the knowledge, motivation, and competences needed to access, understand, appraise, and apply health information in everyday decisions about healthcare, disease prevention, and health promotion (Sørensen *et al*. 2012). Health literacy is not solely an individual cognitive skill set. Social conditions and the demands that organizations place on people also shape whether health information and services are understandable and usable (Nutbeam 2000, Nutbeam and Lloyd 2021, Abel and Benkert 2022, Smith *et al*. 2025). From a life-course perspective, health literacy develops through accumulated educational experiences, material and social conditions, learning opportunities, and encounters with healthcare systems (Clouston *et al*. 2017, Guo *et al*. 2020). By definition, limited health literacy entails difficulty with these activities, for example when navigating services or weighing risks and benefits. It has been associated with poorer self-rated health, less favorable health behaviors, and greater difficulty managing chronic conditions (Marshall *et al*. 2025, Zaghloul *et al*. 2025).

One outcome of particular relevance to health promotion is health-related quality of life (HRQoL), often measured with generic preference-based measures such as the EuroQol five-dimension, five-level instrument (EQ-5D-5L; Herdman *et al*. 2011). HRQoL is important as an outcome in its own right and as an indicator of broader health disadvantage. Poorer HRQoL in general populations has been associated with a higher risk of subsequent mortality (Phyo *et al*. 2020). Among middle-aged and older adults, poorer quality of life has been associated with the presence of multiple chronic conditions and with a greater number of functional limitations (Wilk *et al*. 2024). A systematic review found an overall positive association between health literacy and quality of life (Zheng *et al*. 2018). However, much of the literature focuses on specific patient groups, university students, or older adults (Sayah *et al*. 2016, Rababah *et al*. 2020, Prihanto *et al*. 2021, Marshall *et al*. 2025). Studies in general adult populations in high-income welfare states remain comparatively limited.

Perceived stress may be relevant to the health literacy-HRQoL association. According to the transactional model of stress and coping, stress arises when demands are appraised as taxing or exceeding available resources (Lazarus and Folkman 1984). Perceived stress reflects the extent to which people experience their lives as unpredictable, uncontrollable, and overloaded (Cohen *et al*. 1983, 2007). Health literacy is relevant to both healthcare decision-making and chronic disease self-management (Cabezas *et al*. 2025, Muscat *et al*. 2025). Adults reporting higher perceived stress also tend to report poorer HRQoL and greater depressive symptoms (Serrano *et al*. 2024, Sjöblom *et al*. 2026). These observations make a stress-related indirect association plausible.

Chronic disease burden may be an important context for the association between perceived stress and HRQoL. Multimorbidity has been linked to both higher perceived stress and poorer quality of life (Zhao *et al*. 2023, Wilk *et al*. 2024), suggesting that stress may become especially relevant as disease burden increases. Here, chronic disease burden refers to the number of long-term conditions (Bernell and Howard 2016). Multimorbidity is conventionally defined as the coexistence of two or more long-term conditions (Nicholson *et al*. 2024). Multimorbidity is common in community populations and becomes more prevalent with age (Chowdhury *et al*. 2023). In Finland, it is already common by midlife (Junttila *et al*. 2026) and is socially patterned (Andersén *et al*. 2021). The stress proliferation framework proposes that chronic illnesses can generate secondary strains across work, family life, and everyday functioning (Pearlin *et al*. 2005). Cumulative-risk perspectives further suggest that multiple stressors can combine rather than operate independently (Evans *et al*. 2013). People with multimorbidity may also face substantial treatment burden, including accumulating healthcare tasks and related social, emotional, and financial demands (Lee *et al*. 2024). Perceived stress may therefore be more strongly associated with poorer HRQoL when disease burden is greater.

Health literacy has been examined in relation to multimorbidity and quality of life (Yogesh *et al*. 2024), but few studies have directly tested whether it modifies associations between chronic disease burden and health outcomes. The available studies have reported different patterns across populations and outcomes (Wang *et al*. 2015, Schönenberg and Prell 2025). The present study instead examines whether differences by chronic disease burden appear in the health literacy-perceived stress association, in the perceived stress-HRQoL association, or in both. This distinction matters for health promotion. Differences concentrated in the first association would emphasize health literacy support tailored to disease burden. Differences concentrated in the second would draw attention to stress-sensitive care, treatment burden, and service complexity among people managing several conditions.

Finland offers a useful high-income Nordic setting for this question. Its health system provides universal population coverage and a broad statutory benefits package, but timely access remains uneven, with comparatively high unmet medical needs and long waiting times in publicly funded services (OECD/European Observatory on Health Systems and Policies 2025). Universal entitlement therefore does not remove practical barriers to care. In this setting, a validation study based on the broader PROM 2024 survey reported evidence of a unidimensional structure and largely adequate measurement invariance for the Finnish HLS-Q12 (Zhou *et al*. 2026), supporting use of its composite score in the present study.

Accordingly, this study examined the direct, indirect, and total cross-sectional associations among health literacy, perceived stress, and HRQoL in a national adult sample. It also tested whether the main associations differed across chronic disease-burden groups. All models adjusted for age, gender, education, employment status, and financial strain. Figure 1 summarizes the conceptual model.

**Figure 1:**
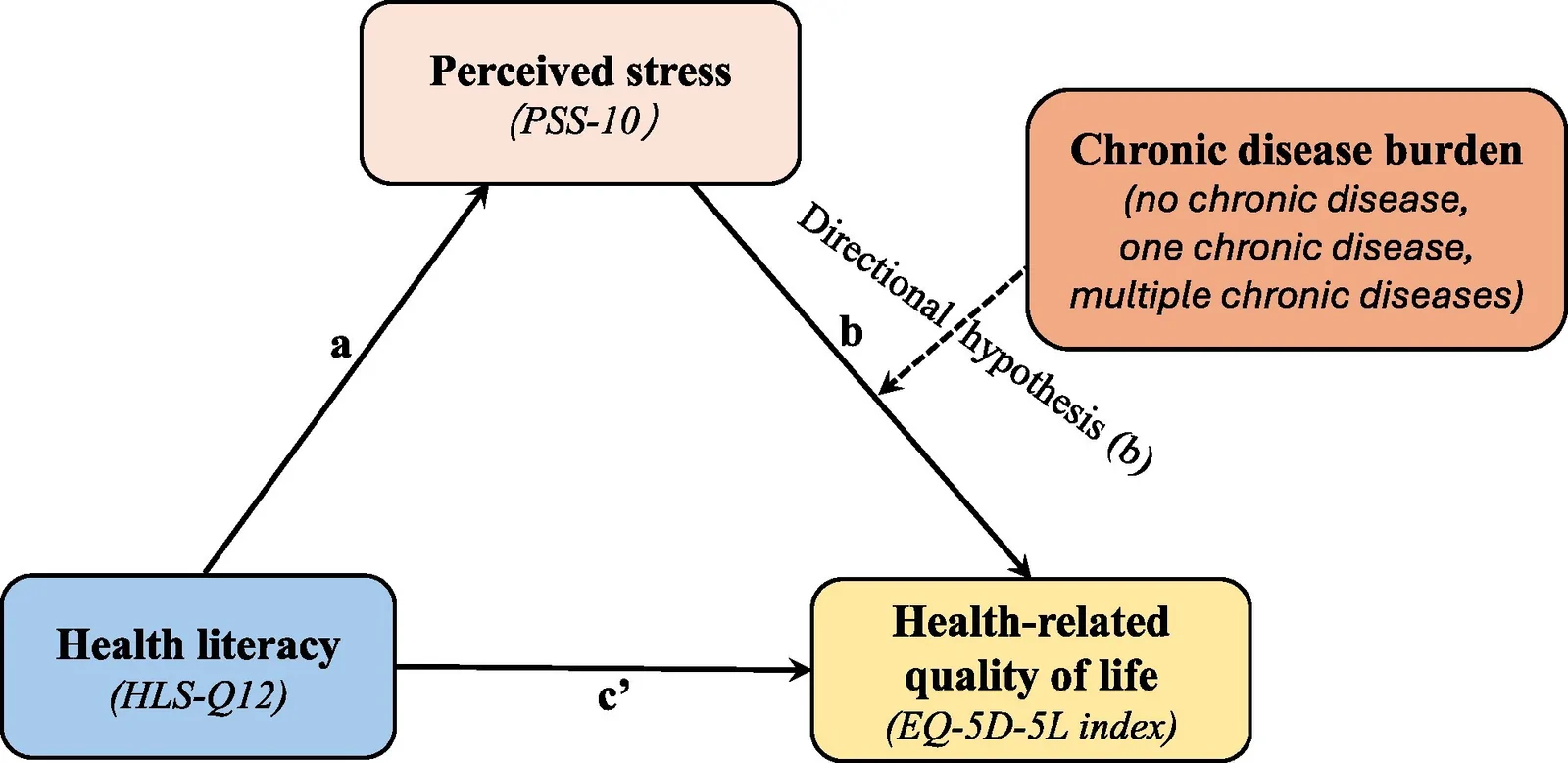
Conceptual model of a stress-related association linking health literacy to HRQoL. Group differences were examined for all three component paths, with a directional hypothesis specified for the perceived stress-HRQoL association. All models adjusted for age, gender, education, employment status, and financial strain.

Hypothesis 1 (H1). Higher health literacy is associated with better HRQoL, including an indirect association through lower perceived stress.

Hypothesis 2 (H2). We hypothesized that the stress-related indirect association between health literacy and HRQoL would differ by chronic disease burden. Based on stress proliferation and cumulative-risk perspectives, we expected the negative association between perceived stress and HRQoL to become progressively stronger across the no-disease, one-disease, and multiple-disease groups. Group differences in the health literacy-perceived stress association and in the remaining direct health literacy-HRQoL association were also examined without directional hypotheses.

## Methods

### Data and participants

The study uses data from the PROM 2024 population survey of adults living in Finland. The survey comprised a nationally recruited panel sample and a supplementary regional sample recruited through additional channels in North Savo outside the national quota design. The present analyses used only the national panel sample. Bilendi recruited this sample from its population panel using quotas for age group, gender, and geographical region designed to approximate the Finnish adult population aged 18 years and older. The survey was administered online from 28 May to 5 July 2024. Participants received a small voucher for completing the questionnaire. A total of 26 600 panel members were invited, of whom 4963 started the survey and 4003 provided final responses. Thus, 18.7% of those invited started the survey and 15.0% provided final responses. Among respondents who started the survey, 960 responses were incomplete or excluded during quality control. The completed national sample comprised 4003 adults aged 19–79 years.

The supplied post-stratification weights were calculated as the ratio of the population proportion to the observed sample proportion within each age-group-by-gender cell. The population reference data were obtained from Statistics Finland and referred to the population as of 31 December 2023. In the full national sample, the weights ranged from 0.767 to 4.895 and had a mean of 1.01.

The study was conducted in accordance with the Declaration of Helsinki. Participants received written study information and provided informed consent before completing the online survey. In accordance with the Finnish National Board on Research Integrity (TENK) guidelines and the procedures of the University of Eastern Finland Committee on Research Ethics, prior ethical review was not required for this study. Data-protection arrangements were reviewed in consultation with the Data Protection Officer at the University of Eastern Finland. Data processing complied with the General Data Protection Regulation and Finnish data-protection legislation. Informed consent was obtained from all individual participants included in the study.

## Measures

### Health literacy

Health literacy was assessed using the 12-item HLS-Q12 (Finbråten *et al*. 2018), with response options ranging from 1 (“very difficult”) to 4 (“very easy”). A composite score was computed by summing all 12 items (range 12–48), with higher values indicating higher health literacy. The Finnish HLS-Q12 used in the PROM 2024 survey was translated from English by bilingual members of the research team proficient in both Finnish and English and subsequently reviewed within the team for clarity and conceptual consistency. A previous validation study supported a unidimensional structure and largely adequate measurement invariance for the Finnish HLS-Q12 (Zhou *et al*. 2026). Internal consistency in the present analytic sample was excellent (Cronbach’s *α* = .901; McDonald’s *ω* = .902).

### Perceived stress

Perceived stress was measured using the 10-item Perceived Stress Scale (PSS-10; Cohen *et al*. 1983, Cohen and Williamson 1988), which assesses the extent to which individuals perceive their lives as unpredictable, uncontrollable and overloaded during the previous month. The Finnish PSS-10 used in PROM 2024 was based on the version previously used in the Finnish StopDia study (Lavikainen *et al*. 2022). Response options ranged from 1 (“never”) to 5 (“very often”), yielding a total score ranging from 10 to 50. Four positively worded items (items 4, 5, 7, 8) were reverse-coded so that higher scores indicated higher perceived stress. Internal consistency was good in our sample (Cronbach’s *α* = .880; McDonald’s *ω* = .883).

### Health-related quality of life

HRQoL was assessed using the official Finnish self-complete digital version of the EQ-5D-5L instrument developed by the EuroQol Group (Herdman *et al*. 2011). The measure consists of five dimensions (mobility, self-care, usual activities, pain/discomfort, and anxiety/depression), each rated on five levels (1 = no problems, 5 = extreme problems). In the present study, respondents’ answers were converted into a single summary index using the EQ-5D-5L value set for England, as no Finnish EQ-5D-5L value set was available (Devlin *et al*. 2018, Penttinen and McLellan 2025). Higher index scores indicate better HRQoL. Values below zero represent health states valued as worse than dead, and 1.00 represents full health. The EQ visual analog scale (EQ-VAS), a global self-rating of current health from 0 to 100, was used as an alternative outcome in a sensitivity analysis.

Chronic disease burden was measured with a checklist of 21 self-reported long-term conditions. Participants indicated whether they had each condition (1 = yes and 0 = no), and the number of reported conditions was summed. Participants were categorized as having no chronic disease, one chronic disease, or multiple chronic diseases (two or more conditions). The complete list of conditions is provided in Supplementary Table S1.

Age was calculated as the survey year (2024) minus the reported year of birth and was treated as a continuous variable. Gender was assessed with four response options: male, female, other, and prefer not to say. For the multivariable models, the binary gender variable used in the weighting scheme was applied, with female as the reference category. Responses “other” and “prefer not to say” were coded as missing. Educational level was assessed using six response categories and recoded into three levels: basic education (comprehensive school only), medium education (upper secondary or post-secondary non-tertiary education, including older college-level diplomas), and high education (university of applied sciences or university). Basic education was the reference category. Employment status was assessed with multiple response categories and recoded into three groups: employed (including employees, senior staff, entrepreneurs, and other paid employees), retired, and other non-employed (including students, unemployed individuals, homemakers, and other temporary non-labor-force statuses). The reference category is employed.

Perceived financial strain was evaluated with an item adapted from the EU statistics on income and living conditions “ability to make ends meet” question. Participants were asked: “Taking into account all household income, how easy or difficult is it to cover your expenses?” Responses ranged from 1 (very easy) to 6 (very difficult), with higher scores indicating greater financial strain (Eurostat 2025).

### Analytic strategy

Weighted descriptive statistics were calculated for the full national sample (*N* = 4003), while weighted Pearson correlations were computed for the complete-case analytic sample (*N* = 3716). Analytic sample construction and missingness are summarized in Supplementary Table S2. Both analyses were conducted in IBM SPSS Statistics 29.0 using the supplied post-stratification weights. Tables 1 and 2 are descriptive. Formal tests relevant to H1 and H2 were conducted in the path models. Internal consistency of the HLS-Q12 and PSS-10 was evaluated with Cronbach’s *α* and McDonald’s *ω* (McNeish 2018). The *ω* was estimated from weighted one-factor congeneric models in the analytic sample (Supplementary Table S3).

**Table 1:** Weighted descriptive characteristics across chronic disease-burden groups.

				Total sample	No chronic disease	One chronic disease	Multiple chronic diseases
Variable	*N*	Weighted *N*	Range	M (SD)	M (SD)	M (SD)	M (SD)
Age	4003	4028	19–79	50.46 (17.88)	40.23 (14.69)	48.05 (17.23)	57.12 (16.86)
HL^a^	3843	3848	12–48	37.57 (5.71)	37.84 (5.61)	37.83 (5.71)	37.28 (5.76)
Perceived stress^b^	3895	3901	10–50	26.75 (6.75)	25.46 (5.80)	26.34 (6.42)	27.65 (7.25)
HRQoL^c^	4003	4028	−0.24–1.00	0.85 (0.16)	0.93 (0.10)	0.88 (0.14)	0.79 (0.18)
Perceived financial strain^d^	4003	4028	1–6	3.29 (1.22)	3.08 (1.12)	3.19 (1.16)	3.45 (1.28)
Variable	*N*	Weighted *N*		%	%	%	%
Gender							
Female	2062	2035		50.8	41.3	49.0	56.9
Male	1916	1967		49.2	58.7	51.0	43.1
Education level							
Basic education	328	373		9.3	5.9	7.8	11.8
Medium education	2119	2103		52.3	49.1	50.5	54.9
High education	1553	1547		38.5	45.0	41.7	33.3
Employment status							
Employed	2342	2115		52.5	70.9	62.8	37.5
Retired	1011	1301		32.3	8.8	23.4	49.4
Other non-employed	647	610		15.2	20.3	13.8	13.1

Descriptive statistics are weighted using post-stratification weights based on age group and gender. *N* refers to unweighted valid cases. Weighted *N* refers to the sum of post-stratification weights among valid cases and may differ across variables. Group sizes (unweighted): no chronic disease, *N* = 1081; one chronic disease, *N* = 1056; multiple chronic diseases, *N* = 1866. Percentages for categorical variables are column percentages within chronic disease-burden groups. ^a^Higher scores indicate better health literacy; ^b^Higher scores indicate higher perceived stress; ^c^England value set; higher scores indicate better health-related quality of life; ^d^Higher scores indicate greater financial strain.

**Table 2:** Weighted correlations among the main study variables.

	M	SD	HL	Perceived stress	HRQoL	Age	Gender	Education
HL	37.53	5.71	–					
Perceived stress	26.73	6.75	−0.342***	–				
HRQoL	0.85	0.16	0.221***	−0.509***	–			
Age	50.11	17.78	0.005	−0.292***	−0.021	–		
Gender	0.49	0.50	−0.144***	−0.082***	0.073***	−0.034*	–	
Education	2.30	0.62	0.133***	−0.151***	0.156***	−0.030	−0.039*	–
Financial strain	3.30	1.22	−0.175***	0.450***	−0.343***	−0.117***	−0.094***	−0.186***

N = 3716 unweighted complete cases. Means, standard deviations, and Pearson correlations were calculated using the supplied post-stratification weights. Gender was coded as 0 = female, 1 = male. Education was treated as an ordinal three-category variable coded 1 = basic, 2 = medium, and 3 = high for the descriptive correlations and was represented by two dummy variables in the path models. M = mean; SD = standard deviation; HL = health literacy; HRQoL = health-related quality of life. * *P* < .05, *** *P* < .001.

Observed-variable path analyses were conducted in Mplus 8.11 using maximum likelihood estimation with robust standard errors (MLR) and the supplied post-stratification weights. The overall model included paths from health literacy to perceived stress, from perceived stress to HRQoL, and directly from health literacy to HRQoL. Age, gender, education (dummy-coded), employment status (dummy-coded), and financial strain were modeled as covariates of perceived stress and HRQoL. Health literacy and perceived stress were mean-centered before estimation. The overall model used full analytic sample means and the multigroup model used group-specific means. Throughout the manuscript, direct, indirect, and total associations refer to a statistical decomposition of cross-sectional associations and do not imply causal effects or temporal ordering (Maxwell and Cole 2007).

Differences by chronic disease burden were examined in multigroup path models across three groups: participants with no chronic disease, one chronic disease, and multiple chronic diseases. All regression paths were freely estimated across groups. MODEL CONSTRAINT was used to define group-specific indirect associations and to test pairwise differences in the health literacy-perceived stress, perceived stress-HRQoL, remaining direct health literacy-HRQoL, and indirect associations. Pairwise tests were based on unstandardized coefficients. Standard errors and 95% confidence intervals for derived parameters were calculated with the delta method under MLR. Statistical significance was evaluated using two-tailed tests at *α* = .05. We report unstandardized coefficients (*B*), robust standard errors, standardized coefficients (*β*), *P*-values, and 95% confidence intervals.

The freely estimated observed-variable path models were just-identified. Accordingly, global fit indices were not used for model evaluation. Models were evaluated using parameter estimates, robust standard errors, confidence intervals, explained variance, and tests of between-group differences. Supplementary Tables S4 and S5 report complete estimates, including covariate paths.

Complete-case analysis was used for the path models, yielding 3716 of 4003 respondents (92.8%). The excluded cases included 25 respondents without a female or male response on the binary analytic gender variable. Missingness was concentrated in the HLS-Q12 and PSS-10 scores, with the HLS-Q12 contributing the largest number of missing values. Because full information maximum likelihood estimation in the specified models would not have retained cases missing the observed exogenous HLS-Q12 score, we used the same complete-case sample for the overall and multigroup models. Supplementary Table S2 reports variable-specific missingness and unweighted comparisons between included and excluded respondents. Excluded respondents were slightly older and differed modestly in education. No other included-versus-excluded comparisons reached statistical significance.

To address possible conceptual proximity between perceived stress and the anxiety/depression dimension of the EQ-5D-5L, we repeated the overall analysis with EQ-VAS as an alternative global health outcome. The sensitivity analysis used the same sample, covariates, weights, and estimator (Supplementary Table S6).

## Results

### Sample characteristics

Weighted sample characteristics are shown in Table 1. In the weighted national sample, 25.7% reported no chronic disease, 25.5% one chronic disease, and 48.7% multiple chronic diseases. Overall, 74.3% reported at least one condition. Participants with multiple chronic diseases were older and, on average, reported slightly lower health literacy, higher perceived stress, lower HRQoL, and greater financial strain than the other groups. Employment was reported by 70.9% of participants with no chronic disease, 62.8% of those with one chronic disease, and 37.5% of those with multiple chronic diseases. The corresponding proportions who were retired were 8.8%, 23.4%, and 49.4%.

### Correlations

Table 2 presents correlations among study variables in the analytic sample. Health literacy correlated negatively with perceived stress and positively with HRQoL. Perceived stress showed the strongest bivariate correlation with HRQoL. Age correlated negatively with perceived stress but showed near-zero correlations with health literacy and HRQoL. Among the covariates, financial strain had the strongest correlations with perceived stress and HRQoL. Females reported higher health literacy and perceived stress but lower HRQoL than males, although these correlations were modest.

### Overall path model

Table 3 summarizes the results of the overall path model, with complete covariate estimates reported in Supplementary Table S4. Higher health literacy was associated with lower perceived stress (*β* = −.286, *P* < .001), and higher perceived stress was associated with poorer HRQoL (*β* = −.477, *P* < .001). The remaining direct health literacy-HRQoL association was small and not statistically significant. The indirect association through perceived stress was statistically significant (*β* = .137, *P* < .001) and represented 82.4% of the total association based on unstandardized coefficients (95% CI, 64.2%–100.7%). The total association was significant, and these findings were consistent with H1. The model explained 34.8% of variance in perceived stress and 32.0% of variance in HRQoL.

**Table 3:** Direct, indirect, and total associations among health literacy, perceived stress, and HRQoL.

Association	*B*	SE (*B*)	95% CI	*β*	*P*-value
Health literacy → perceived stress	−0.338	0.019	[−0.374, −0.302]	−.286	<.001
Perceived stress → HRQoL	−0.011	0.001	[−0.012, −0.010]	−.477	<.001
Direct: health literacy → HRQoL	0.001	0.000	[0.000, 0.002]	.029	.108
Indirect: health literacy → perceived stress → HRQoL	0.004	0.000	[0.003, 0.004]	.137	<.001
Total: health literacy → HRQoL	0.005	0.001	[0.004, 0.006]	.166	<.001

*N* = 3716. All analyses used MLR and post-stratification weights. Models adjusted for age, gender, education, employment status, and perceived financial strain. HL = health literacy (HLS-Q12, 12–48); stress = perceived stress (PSS-10, 10–50); HRQoL = health-related quality of life (EQ-5D-5L index, England value set). *B* = unstandardized coefficient; *β* = standardized coefficient (STDYX); SE = standard error; CI = confidence interval. Direct association adjusted for perceived stress. Total association = direct association + indirect association. The indirect-to-total association ratio was 82.4% (95% CI, 64.2%–100.7%), calculated from unrounded unstandardized coefficients. *R*^2^ = 34.8% for perceived stress and 32.0% for HRQoL.

### Multigroup path model

Table 4 exhibits the multigroup results with full details in Supplementary Table S5. Figure 2 displays the group-specific perceived stress-HRQoL slopes. On the unstandardized metric, the perceived stress-HRQoL association became progressively more negative across the no-disease, one-disease, and multiple-disease groups (*B* = −0.006, −0.008, and −0.012, respectively). All three pairwise differences were statistically significant. This pattern was consistent with H2.

**Figure 2:**
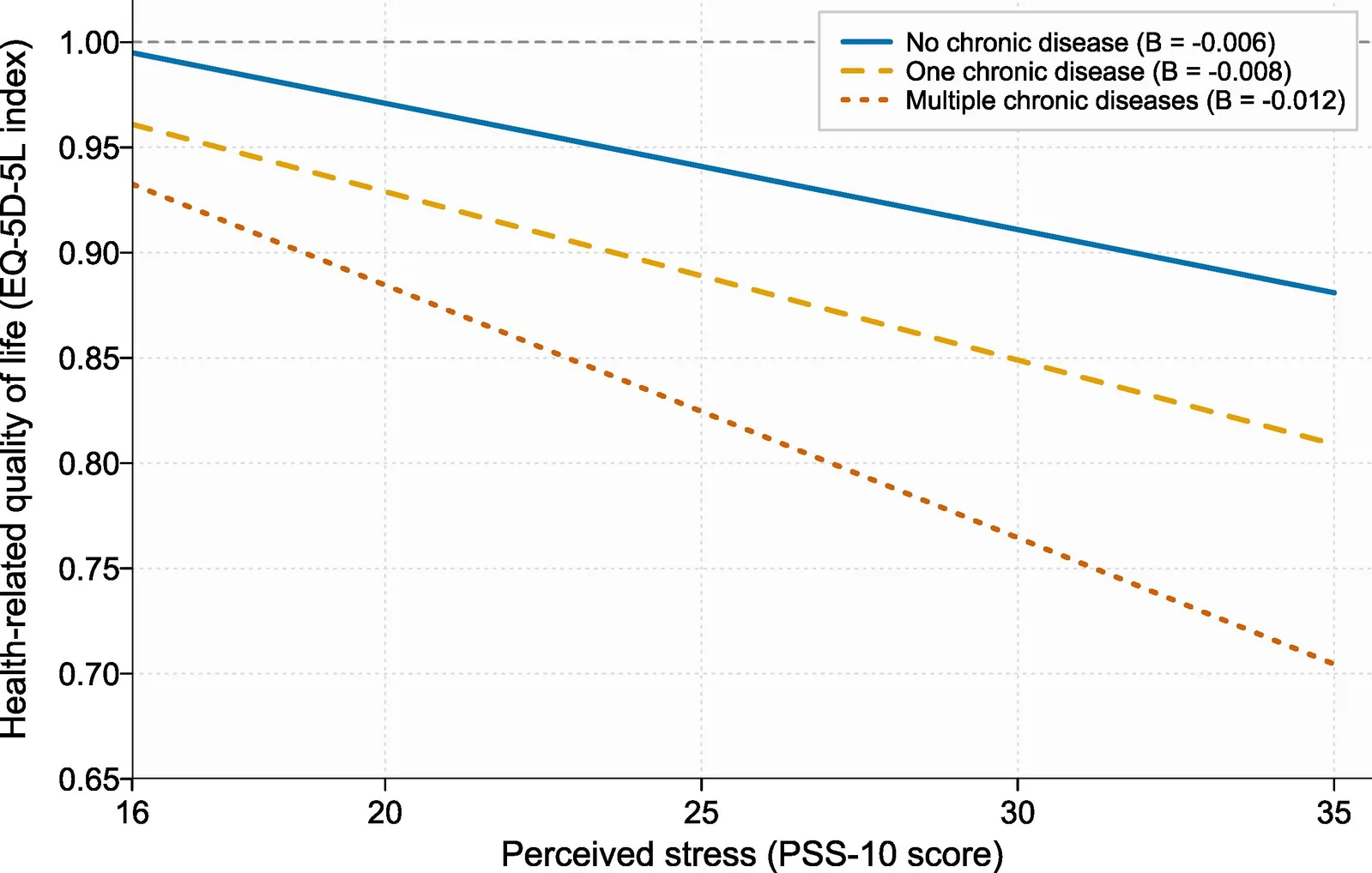
Association between perceived stress and health-related quality of life by chronic disease burden. Lines show the estimated association between perceived stress and HRQoL across chronic disease-burden groups (*N* = 3716). The unstandardized slopes were *B* = −0.006 for no chronic disease, *B* = −0.008 for one chronic disease, and *B* = −0.012 for multiple chronic diseases. All slopes were statistically significant (*P* < .001), and pairwise differences between all groups were statistically significant. Health literacy and covariates were held at their group-specific weighted means.

**Table 4:** Multigroup path analysis across chronic disease-burden groups.

Path	No disease (*N* = 1008)	One disease (*N* = 984)	Multiple diseases (*N* = 1724)	Pairwise group differences
	*B* (*β*)	*B* (*β*)	*B* (*β*)	
a. Health literacy → perceived stress	−0.310 (−0.296)***	−0.345 (−0.306)***	−0.337 (−0.269)***	All *P* ≥ .44
b. Perceived stress → HRQoL	−0.006 (−0.410)***	−0.008 (−0.370)***	−0.012 (−0.481)***	All *P* ≤ .032
c´. Direct: health literacy → HRQoL	0.000 (0.018)	0.002 (0.069)	0.001 (0.047)	All *P* ≥ .171
ab. Indirect: health literacy → perceived stress → HRQoL	0.002 (0.122)***	0.003 (0.113)***	0.004 (0.130)***	All *P* ≤ .036
*R* ^2^ (stress)	27.5%	37.0%	40.7%	–
*R* ^2^ (HRQoL)	20.3%	22.0%	33.9%	–

*N* = 3716. Estimates are from a weighted multigroup path model adjusted for age, gender, education, employment status, and financial strain. HRQoL = health-related quality of life (EQ-5D-5L); *B* = unstandardized coefficient; *β* = standardized coefficient (STDYX). Health literacy and perceived stress were mean-centered within chronic disease-burden groups. Significance markers and pairwise group-difference tests are based on the unstandardized coefficients. Values displayed as 0.000 reflect rounding to three decimal places. Complete pairwise comparisons and covariate estimates are reported in Supplementary Table S5, together with a descriptive summary of the EQ-5D-5L ceiling distribution. *** *P* < .001.

The health literacy-perceived stress association and the remaining direct health literacy-HRQoL association did not differ statistically across groups. On the unstandardized metric, the indirect associations through perceived stress were larger with greater disease burden, and all pairwise differences were statistically significant. Differences in the component paths were therefore concentrated in the perceived stress-HRQoL association. Explained variance ranged from 27.5% to 40.7% for perceived stress and from 20.3% to 33.9% for HRQoL.

The proportion of participants at the upper bound of the EQ-5D-5L index was 46.1% in the no-disease group, 23.7% in the one-disease group, and 8.4% in the multiple-disease group in the weighted analytic sample (Supplementary Table S5, Panel E).

### Sensitivity analysis

When EQ-VAS replaced the EQ-5D-5L index, the standardized indirect association was similar to that in the primary model (*β* = .130 versus *β* = .137). The indirect-to-total association ratio was smaller at 63.3% (95% CI, 50.2%–76.4%), compared with 82.4% in the primary model. Meanwhile, a small direct association between health literacy and EQ-VAS was observed (*β* = .075, *P* < .001). Supplementary Table S6 reports the complete results.

## Discussion

This study examined whether perceived stress formed an indirect statistical link between health literacy and HRQoL and whether the main associations differed by chronic disease burden. Higher health literacy was associated with lower perceived stress, and higher perceived stress was associated with poorer HRQoL. The indirect association through perceived stress represented most of the modest total health literacy-HRQoL association, while the remaining direct association was small and not statistically significant. Across chronic disease-burden groups, differences in the component paths were concentrated in the perceived stress-HRQoL association. The health literacy-perceived stress association was similar across groups. These results were consistent with H1 and H2 on the unstandardized metric. The principal contribution lies in identifying where the group difference appeared within the fitted model.

The association between higher health literacy and lower perceived stress is consistent with the transactional model of stress and coping. This model emphasizes how people appraise demands in relation to the resources available to manage them (Lazarus and Folkman 1984). Health literacy may operate as both an informational and a psychosocial resource. Greater ability to access, understand, appraise, and use health information may reduce uncertainty when people make health decisions or navigate services. It may also support a stronger sense of control in health-related situations (Nutbeam 2000, Paasche-Orlow and Wolf 2007). Our findings extend previous evidence on health literacy and HRQoL (Zheng *et al*. 2018, Ehmann *et al*. 2020) by showing a distinct stress-related statistical pattern in a general adult population.

The large indirect-to-total association ratio should be interpreted in relation to the modest total association. The indirect association represented 82.4% of the total association based on unstandardized coefficients, but this value should not be interpreted as the proportion of a causal mechanism. The ratio was large partly because the remaining direct association was close to zero, and its wide confidence interval indicates uncertainty around the exact proportion. In the EQ-VAS sensitivity analysis, the standardized indirect association was similar, while the indirect-to-total ratio was lower and a small direct association remained. This suggests that the broader stress-related pattern did not depend entirely on the anxiety/depression dimension of the EQ-5D-5L, although the estimated proportion varied with the outcome measure. EQ-VAS is also self-reported, so common-method variance and shared psychological appraisal remain possible.

The multigroup findings are consistent with stress proliferation theory, which proposes that chronic health problems can generate additional strains across work, family life, treatment routines, and everyday functioning (Pearlin *et al*. 2005). They also accord with cumulative-risk perspectives, which emphasize that multiple demands can become more consequential when they accumulate (Evans *et al*. 2013). Managing several long-term conditions may involve more appointments, medication routines, care coordination, functional restrictions, and competing treatment demands. These demands can extend beyond healthcare tasks to social, emotional, and financial burdens, which may help explain why perceived stress was more strongly associated with poorer HRQoL among adults with multiple conditions (Lee *et al*. 2024). Burden of Treatment Theory provides a complementary explanation by focusing on the balance between treatment workload and the capacity and resources available to manage it (May *et al*. 2014, Smyth *et al*. 2025). The cumulative complexity model similarly highlights how this balance may become increasingly difficult to sustain as health and treatment demands accumulate (Shippee *et al*. 2012, Zhao *et al*. 2025).

Some previous studies have examined whether health literacy modifies associations between chronic disease or multimorbidity and health outcomes, with findings varying across populations and outcomes (Wang *et al*. 2015, Schönenberg and Prell 2025). The present study instead examined where differences by chronic disease burden appeared within the health literacy-stress-HRQoL model. The group pattern emerged in the perceived stress-HRQoL association, while the health literacy-perceived stress association remained similar across groups. This suggests that greater disease burden was not accompanied by a stronger association between health literacy and perceived stress. Rather, perceived stress was more closely associated with HRQoL when disease burden was greater. The findings therefore extend previous moderation research by locating the observed heterogeneity within a specific part of the fitted model.

The magnitude of the group differences also depends on the coefficient metric and the distribution of HRQoL. On the unstandardized metric, the perceived stress-HRQoL association was approximately twice as large in the multiple-disease group as in the no-disease group. The standardized coefficients showed a less monotonic pattern because standardization incorporates group-specific variation in perceived stress and HRQoL. The EQ-5D-5L distribution provides further context. Almost half of participants without chronic disease were at the upper bound, compared with substantially smaller proportions in the two disease groups. Greater compression near the upper bound may have contributed to the shallower unstandardized slope in the no-disease group. The observed gradient should therefore be interpreted in light of both group differences in the association and differences in the outcome distribution.

The indirect association was modest on the EQ-5D-5L scale. In the multiple-disease group, a one-standard-deviation difference in health literacy corresponded to ∼0.02 index points. This provides a scale-based indication of magnitude rather than an estimate of the change produced by increasing health literacy. Minimally important differences for the EQ-5D-5L vary across populations, conditions, and baseline health states, so no single threshold provides a firm basis for judging clinical importance in this general adult sample (Cheng *et al*. 2024). The result is therefore more informative about the pattern and location of the association than about clinically meaningful individual change. Future studies should determine whether these differences persist over time and have practical relevance.

### Strengths and limitations

This study has several strengths. First, we used a large national adult sample with post-stratification weights based on age group and gender. The main constructs were assessed with established instruments, and reliability was evaluated using both Cronbach’s *α* and McDonald’s *ω*. The multigroup analysis identified where differences by chronic disease burden appeared within the fitted model. The EQ-VAS sensitivity analysis also addressed possible content overlap between perceived stress and the anxiety/depression dimension of the EQ-5D-5L. Complete covariate estimates and pairwise group contrasts are provided in the Supplementary material.

Several limitations should also be considered. First, the cross-sectional design does not establish temporal ordering or causal direction. Greater stress may be associated with poorer HRQoL, but poorer health and greater disease burden may also increase perceived stress. Stress and ill health may also affect how people process and respond to health information.

Second, all key variables were self-reported, which may introduce common-method variance. Perceived stress and the anxiety/depression dimension of the EQ-5D-5L also have some conceptual proximity. The EQ-VAS sensitivity analysis addressed this specific concern but did not remove shared self-report and appraisal processes.

Third, our measure of chronic disease burden was based on a self-reported count of long-term conditions. The resulting count did not distinguish condition type, severity, duration, functional limitation, treatment burden, or specific combinations of conditions. The checklist was broad and included common conditions such as high blood pressure, high cholesterol, migraine, and back problems. This breadth helps explain the high proportion reporting at least one condition and limits direct comparison with studies using narrower condition lists. Future studies could examine whether severity, functional limitation, treatment burden, or specific condition clusters provide a more informative account of differences in the perceived stress-HRQoL association.

Fourth, recruitment through a quota-based online panel was not probability sampling. Post-stratification weights aligned the sample with the Finnish adult population by age group and gender but did not adjust for education or other characteristics. Complete-case analysis excluded 7.2% of respondents, and excluded participants differed modestly in age and education. Adults aged 80 years or older were not represented. The path models also used the binary gender variable applied in the weighting scheme, so respondents without a female or male value were not included in these models.

Fifth, we used the England value set for the EQ-5D-5L (Devlin *et al*. 2018) because no Finnish EQ-5D-5L value set was available (Penttinen and McLellan 2025). Health literacy and perceived stress were analyzed as observed composite scores, and measurement invariance was not examined specifically across chronic disease-burden groups. Future work should assess measurement comparability across disease-burden groups and test whether the findings persist in latent-variable models.

Finally, chronic disease burden was the only grouping variable considered. Other potential sources of heterogeneity, including language proficiency, migration background, caregiving responsibilities and geographic location, remain to be examined. Future studies could examine whether similar patterns are observed across these population groups.

### Implications for health promotion

These findings provide a rationale for approaches that address health literacy, stress, and organizational demands together. Accessible communication, navigation support, and coordinated care may reduce some of the informational and practical demands involved in managing health needs. Reviews suggest that health literacy interventions can improve both health literacy and some health behaviors, while evidence for broader health outcomes remains mixed (Walters *et al*. 2020, Marshall *et al*. 2025). Stress-management support may help people manage psychological demands, but it does not remove confusing information, fragmented services, or treatment workload. Clearer communication may also have limited value when people face substantial financial or health-related pressures. Approaches that combine these components therefore warrant prospective evaluation, particularly among adults managing multiple long-term conditions.

An organizational health literacy perspective extends this implication beyond individual skills. Health literacy also depends on how understandable and usable health information, services, and care processes are (Brach *et al*. 2012, Santana *et al*. 2021). Health-literate organizations can reduce unnecessary demands through clear communication, easier navigation, and better coordination across services. This perspective is relevant in Finland, where universal entitlement coexists with long waits in publicly funded services and differences in access between public and occupational healthcare (OECD/European Observatory on Health Systems and Policies 2025). These organizational features offer a concrete focus for health promotion.

The social patterns observed in the data are consistent with this broader perspective. In the bivariate correlations, perceived financial strain showed the strongest associations among the covariates with stress and HRQoL. This suggests that communication and navigation support may need to be connected with benefits advice, debt counseling, or other forms of social support where appropriate (Samuel *et al*. 2025). Employment was less common among adults with multiple chronic diseases. This pattern may reflect differences in age, functional capacity, health-related movement out of work, or workplace barriers, although the present study cannot distinguish among these explanations. Women reported higher health literacy, a pattern consistent with reviews of gender differences in health literacy (Chakraverty *et al*. 2022, Gonçalves-Fernández and Pino-Juste 2025). They also reported higher perceived stress and poorer HRQoL. Differences in social roles, care responsibilities, health exposures, service use, and reporting processes may contribute to this pattern. Taken together, these patterns indicate that support focused on individual health literacy is unlikely on its own to address the social and organizational conditions associated with stress and quality of life.

## Conclusion

In this national cross-sectional study of Finnish adults, higher health literacy was associated with lower perceived stress and better HRQoL. The indirect association through perceived stress represented a substantial share of the total association, although its estimated size differed across the outcome measures and the cross-sectional design precludes causal interpretation. Differences by chronic disease burden were concentrated in the perceived stress-HRQoL association, which was strongest among adults with multiple chronic diseases. Longitudinal and intervention studies should examine whether service-level approaches combining accessible communication, stress-sensitive support, and reduced treatment burden can improve HRQoL in this group.

## Supplementary Material

daag157_Supplementary_Data

## Data Availability

Due to the sensitive nature of the survey data and the assurances of confidentiality given to participants, the raw data are not publicly available. Access to the data may be granted for qualified research purposes through the study data custodian, subject to the applicable approvals and data use conditions. The corresponding author can provide information on the access request process and can share the analysis code and non-identifiable study materials upon reasonable request.
