# Supplementary material for: Health literacy, perceived stress, and health-related quality of life in Finnish adults: differences by chronic disease burden
Source: Health Promot Int. 2026 Oct 21;41(5):daag157. doi: 10.1093/heapro/daag157 (PMC13684275; doi:10.1093/heapro/daag157)
Supplement: Supplementary file 1 [file daag157_supplementary_data.docx]

# Supplementary Tables

**Supplementary Table S1. Self-reported long-term conditions included in the chronic disease checklist**

| No. | Condition |
| --- | --- |
| 1 | High blood pressure |
| 2 | High cholesterol |
| 3 | Depression |
| 4 | Other mental health problem |
| 5 | Memory disorder or dementia |
| 6 | Asthma |
| 7 | Chronic obstructive pulmonary disease |
| 8 | Type 1 diabetes |
| 9 | Type 2 diabetes |
| 10 | Hypothyroidism or other thyroid disorder |
| 11 | Migraine or other chronic headache |
| 12 | Skin disease |
| 13 | Heart disease |
| 14 | Stroke |
| 15 | Gastrointestinal disease |
| 16 | Eye disease |
| 17 | Rheumatic disease |
| 18 | Spinal degeneration, sciatica, or other back condition |
| 19 | Knee or hip osteoarthritis |
| 20 | Cancer |
| 21 | Other long-term illness (please specify) |

***Note.*** Respondents were asked, “Do you have one or more of the following long-term (chronic) illnesses?” and responded yes or no to each condition. The number of reported conditions was summed and categorized as no chronic disease, one chronic disease, or multiple chronic diseases, defined as two or more reported conditions.

**Supplementary Table S2. Analytic sample construction and comparison of included and excluded participants**

**Supplementary Table S2. Panel A.** **Analytic sample construction**

| Item | N | % of total sample |
| --- | --- | --- |
| Completed national sample | 4,003 | 100.0 |
| Included in analytic sample | 3,716 | 92.8 |
| Excluded from analytic sample | 287 | 7.2 |

***Note.*** The analytic sample included respondents with complete data on all variables used in the main path models*.*

**Supplementary Table S2. Panel B. Missing or excluded values by model variable**

| Variable | Observed N | Missing or excluded N | Missing or excluded % |
| --- | --- | --- | --- |
| HLS-Q12 total score | 3,843 | 160 | 4.0 |
| PSS-10 total score | 3,895 | 108 | 2.7 |
| EQ-5D-5L index, England value set | 4,003 | 0 | 0.0 |
| Age | 4,003 | 0 | 0.0 |
| Gender category used in weighted model | 3,978 | 25 | 0.6 |
| Medium education dummy | 4,000 | 3 | 0.1 |
| High education dummy | 4,000 | 3 | 0.1 |
| Retired dummy | 4,000 | 3 | 0.1 |
| Other non-employed dummy | 4,000 | 3 | 0.1 |
| Financial strain | 4,003 | 0 | 0.0 |
| Chronic disease burden | 4,003 | 0 | 0.0 |
| Post-stratification weight | 4,003 | 0 | 0.0 |

***Note.*** Missing categories were not mutually exclusive; therefore, variable-specific missing counts do not sum to the total number excluded.

**Supplementary Table S2. Panel C. Number of missing model variables per respondent**

| Number of missing model variables | n | % |
| --- | --- | --- |
| 0 | 3,716 | 92.8 |
| 1 | 270 | 6.7 |
| 2 | 16 | 0.4 |
| 3 | 1 | 0.0 |
| Total | 4,003 | 100.0 |

***Note.*** Gender responses outside the binary categories used in the weighting scheme were treated as missing in the multivariable path models.

**Supplementary Table S2. Panel D. Comparison of included and excluded participants**

| Variable | Excluded from analytic sample | Included in analytic sample | Test statistic | p |
| --- | --- | --- | --- | --- |
| Age, mean (SD) | 50.29 (16.61) | 48.06 (15.92) | Welch F = 4.82 | .029 |
| HLS-Q12 total score, mean (SD) | 38.33 (5.90) | 37.51 (5.71) | Welch F = 2.39 | .124 |
| PSS-10 total score, mean (SD) | 27.40 (6.94) | 27.00 (6.78) | Welch F = 0.55 | .461 |
| EQ-5D-5L index, mean (SD) | 0.83 (0.20) | 0.85 (0.16) | Welch F = 2.81 | .094 |
| Financial strain, mean (SD) | 3.23 (1.29) | 3.36 (1.22) | Welch F = 2.59 | .109 |
| Female, % | 49.2 | 52.0 | chi-square = 0.76 | .384 |
| Male, % | 50.8 | 48.0 |  |  |
| Basic education, % | 12.0 | 7.9 | chi-square = 6.16 | .046 |
| Medium education, % | 52.5 | 53.0 |  |  |
| High education, % | 35.6 | 39.1 |  |  |
| Employed, % | 53.9 | 58.9 | chi-square = 5.95 | .051 |
| Retired, % | 31.3 | 24.8 |  |  |
| Other non-employed, % | 14.8 | 16.3 |  |  |
| No chronic disease, % | 25.4 | 27.1 | chi-square = 1.02 | .600 |
| One chronic disease, % | 25.1 | 26.5 |  |  |
| Multiple chronic diseases, % | 49.5 | 46.4 |  |  |

***Note.*** Percentages are column percentages within included and excluded groups. Continuous-variable comparisons used Welch's F-tests. Categorical-variable comparisons used Pearson's chi-square tests. Comparisons in Panel D were conducted on unweighted data.

**Supplementary Table S3. Reliability estimates for the HLS-Q12 and PSS-10 in the analytic sample**

| Scale | Number of items | Score range | Cronbach's α | McDonald's ω [95% CI] | Analytic sample |
| --- | --- | --- | --- | --- | --- |
| HLS-Q12 | 12 | 12-48 | .901 | .902 [.896, .908] | 3,716 |
| PSS-10 | 10 | 10-50 | .880 | .883 [.875, .891] | 3,716 |

***Note.*** Reliability estimates were calculated in the main analytic sample. McDonald's ω was estimated from weighted one-factor congeneric models using post-stratification weights. Items were coded so that higher scores indicated higher health literacy for the HLS-Q12 and higher perceived stress for the PSS-10.

**Supplementary Table S4. Complete estimates from the overall path model**

| Outcome | Predictor / association | B | SE | 95% CI | β | p |
| --- | --- | --- | --- | --- | --- | --- |
| Perceived stress | Health literacy | -0.338 | 0.019 | [-0.374, -0.302] | -0.286 | < .001 |
| Perceived stress | Age | -0.102 | 0.008 | [-0.118, -0.085] | -0.268 | < .001 |
| Perceived stress | Male (vs. female) | -1.360 | 0.199 | [-1.751, -0.970] | -0.101 | < .001 |
| Perceived stress | Medium education (vs. basic) | -0.652 | 0.360 | [-1.358, 0.053] | -0.048 | .070 |
| Perceived stress | High education (vs. basic) | -1.226 | 0.383 | [-1.976, -0.475] | -0.089 | .001 |
| Perceived stress | Retired (vs. employed) | 0.406 | 0.307 | [-0.196, 1.008] | 0.028 | .186 |
| Perceived stress | Other non-employed (vs. employed) | 0.403 | 0.284 | [-0.153, 0.960] | 0.022 | .155 |
| Perceived stress | Financial strain | 1.914 | 0.098 | [1.721, 2.106] | 0.346 | < .001 |
| HRQoL | Perceived stress | -0.011 | 0.001 | [-0.012, -0.010] | -0.477 | < .001 |
| HRQoL | Health literacy | 0.001 | 0.000 | [0.000, 0.002] | 0.029 | .108 |
| HRQoL | Age | -0.001 | 0.000 | [-0.001, 0.000] | -0.080 | .001 |
| HRQoL | Male (vs. female) | 0.006 | 0.005 | [-0.003, 0.016] | 0.020 | .177 |
| HRQoL | Medium education (vs. basic) | 0.002 | 0.010 | [-0.018, 0.022] | 0.006 | .840 |
| HRQoL | High education (vs. basic) | 0.013 | 0.010 | [-0.006, 0.033] | 0.042 | .176 |
| HRQoL | Retired (vs. employed) | -0.051 | 0.007 | [-0.065, -0.036] | -0.150 | < .001 |
| HRQoL | Other non-employed (vs. employed) | -0.018 | 0.007 | [-0.031, -0.005] | -0.041 | .008 |
| HRQoL | Financial strain | -0.016 | 0.002 | [-0.021, -0.011] | -0.126 | < .001 |
| Decomposition of HL-HRQoL association | Total association | 0.005 | 0.001 | [0.004, 0.006] | 0.166 | < .001 |
| Decomposition of HL-HRQoL association | Indirect association through perceived stress | 0.004 | 0.000 | [0.003, 0.004] | 0.137 | < .001 |
| Decomposition of HL-HRQoL association | Direct association | 0.001 | 0.000 | [0.000, 0.002] | 0.029 | .108 |

*Note.* N = 3,716. Models used post-stratification weights based on age group and gender. HRQoL was measured using the EQ-5D-5L index based on the England value set. Health literacy and perceived stress were grand-mean centered. B = unstandardized coefficient; SE = robust standard error; CI = confidence interval; β = standardized coefficient (STDYX). The indirect association represented 82.4% of the total association based on unstandardized coefficients, 95% CI [64.2%, 100.7%]. R² = .348 for perceived stress and .320 for HRQoL. Because the EQ-5D-5L index ranges from approximately 0 to 1, several unstandardized coefficients are small; values displayed as 0.000 reflect rounding to three decimals. Because the observed-variable path model was just-identified, global fit indices were not substantively interpreted.

**Supplementary Table S5. Multigroup path model by chronic disease burden:**

**Supplementary Table S5. Panel A. Core path estimates**

| Path | Group | B | SE | 95% CI | β | p |
| --- | --- | --- | --- | --- | --- | --- |
| HL -> perceived stress | No chronic disease | -0.310 | 0.031 | [-0.371, -0.249] | -0.296 | < .001 |
| HL -> perceived stress | One chronic disease | -0.345 | 0.033 | [-0.409, -0.281] | -0.306 | < .001 |
| HL -> perceived stress | Multiple chronic diseases | -0.337 | 0.028 | [-0.392, -0.282] | -0.269 | < .001 |
| Perceived stress -> HRQoL | No chronic disease | -0.006 | 0.000 | [-0.007, -0.005] | -0.410 | < .001 |
| Perceived stress -> HRQoL | One chronic disease | -0.008 | 0.001 | [-0.009, -0.006] | -0.370 | < .001 |
| Perceived stress -> HRQoL | Multiple chronic diseases | -0.012 | 0.001 | [-0.013, -0.010] | -0.481 | < .001 |
| HL -> HRQoL, direct association | No chronic disease | 0.000 | 0.001 | [-0.001, 0.001] | 0.018 | .619 |
| HL -> HRQoL, direct association | One chronic disease | 0.002 | 0.001 | [0.000, 0.003] | 0.069 | .054 |
| HL -> HRQoL, direct association | Multiple chronic diseases | 0.001 | 0.001 | [0.000, 0.003] | 0.047 | .079 |
| HL -> perceived stress -> HRQoL, indirect association | No chronic disease | 0.002 | 0.000 | [0.001, 0.002] | -- | < .001 |
| HL -> perceived stress -> HRQoL, indirect association | One chronic disease | 0.003 | 0.000 | [0.002, 0.003] | -- | < .001 |
| HL -> perceived stress -> HRQoL, indirect association | Multiple chronic diseases | 0.004 | 0.000 | [0.003, 0.005] | -- | < .001 |

***Note.*** N = 3,716. Group sizes were n = 1,008 for no chronic disease, n = 984 for one chronic disease, and n = 1,724 for multiple chronic diseases. Models used post-stratification weights based on age group and gender and adjusted for age, gender, education, employment status, and financial strain. Standardized coefficients for the conditional indirect associations are reported in Table 4. Values displayed as 0.000 reflect rounding to three decimals.

**Supplementary Table S5. Panel B. Pairwise differences in focal paths and indirect associations**

| Difference | Estimate | SE | 95% CI | p |
| --- | --- | --- | --- | --- |
| HL -> perceived stress: one disease vs no disease | -0.035 | 0.045 | [-0.123, 0.054] | .440 |
| HL -> perceived stress: multiple diseases vs no disease | -0.027 | 0.042 | [-0.109, 0.055] | .520 |
| HL -> perceived stress: multiple diseases vs one disease | 0.008 | 0.043 | [-0.077, 0.092] | .855 |
| Perceived stress -> HRQoL: one disease vs no disease | -0.002 | 0.001 | [-0.004, 0.000] | .032 |
| Perceived stress -> HRQoL: multiple diseases vs no disease | -0.006 | 0.001 | [-0.008, -0.004] | < .001 |
| Perceived stress -> HRQoL: multiple diseases vs one disease | -0.004 | 0.001 | [-0.006, -0.002] | .001 |
| HL -> HRQoL direct: one disease vs no disease | 0.001 | 0.001 | [-0.001, 0.003] | .171 |
| HL -> HRQoL direct: multiple diseases vs no disease | 0.001 | 0.001 | [-0.001, 0.003] | .225 |
| HL -> HRQoL direct: multiple diseases vs one disease | 0.000 | 0.001 | [-0.002, 0.002] | .886 |
| Indirect association: one disease vs no disease | 0.001 | 0.000 | [0.000, 0.002] | .036 |
| Indirect association: multiple diseases vs no disease | 0.002 | 0.000 | [0.001, 0.003] | < .001 |
| Indirect association: multiple diseases vs one disease | 0.001 | 0.001 | [0.000, 0.002] | .026 |

***Note.*** Pairwise differences were estimated using MODEL CONSTRAINT in Mplus. Negative estimates for the perceived stress–HRQoL path indicate a more negative association in the group named first.

**Supplementary Table S5. Panel C. Explained variance**

| Outcome | No chronic disease | One chronic disease | Multiple chronic diseases |
| --- | --- | --- | --- |
| Perceived stress, R² | .275 | .370 | .407 |
| HRQoL, R² | .203 | .220 | .339 |

***Note.*** R² values are from weighted multigroup path models.

**Supplementary Table S5. Panel D. Covariate estimates by chronic disease burden group**

| Group | Outcome | Covariate | B | SE | β | p |
| --- | --- | --- | --- | --- | --- | --- |
| No chronic disease | Perceived stress | Age | -0.085 | 0.014 | -0.213 | < .001 |
| No chronic disease | Perceived stress | Male (vs. female) | -0.995 | 0.325 | -0.085 | .002 |
| No chronic disease | Perceived stress | Medium education (vs. basic) | -1.967 | 0.769 | -0.170 | .011 |
| No chronic disease | Perceived stress | High education (vs. basic) | -2.549 | 0.785 | -0.219 | .001 |
| No chronic disease | Perceived stress | Retired (vs. employed) | -0.758 | 0.742 | -0.036 | .307 |
| No chronic disease | Perceived stress | Other non-employed (vs. employed) | -0.528 | 0.424 | -0.037 | .213 |
| No chronic disease | Perceived stress | Financial strain | 1.498 | 0.155 | 0.286 | < .001 |
| No chronic disease | HRQoL | Age | -0.001 | 0.000 | -0.136 | .005 |
| No chronic disease | HRQoL | Male (vs. female) | 0.009 | 0.005 | 0.054 | .055 |
| No chronic disease | HRQoL | Medium education (vs. basic) | -0.011 | 0.011 | -0.068 | .335 |
| No chronic disease | HRQoL | High education (vs. basic) | -0.006 | 0.011 | -0.035 | .611 |
| No chronic disease | HRQoL | Retired (vs. employed) | -0.001 | 0.010 | -0.005 | .886 |
| No chronic disease | HRQoL | Other non-employed (vs. employed) | -0.006 | 0.006 | -0.032 | .334 |
| No chronic disease | HRQoL | Financial strain | -0.005 | 0.002 | -0.076 | .006 |
| One chronic disease | Perceived stress | Age | -0.125 | 0.014 | -0.330 | < .001 |
| One chronic disease | Perceived stress | Male (vs. female) | -0.559 | 0.359 | -0.043 | .120 |
| One chronic disease | Perceived stress | Medium education (vs. basic) | 0.192 | 0.567 | 0.015 | .735 |
| One chronic disease | Perceived stress | High education (vs. basic) | -0.456 | 0.562 | -0.035 | .416 |
| One chronic disease | Perceived stress | Retired (vs. employed) | 0.415 | 0.564 | 0.027 | .462 |
| One chronic disease | Perceived stress | Other non-employed (vs. employed) | 0.710 | 0.510 | 0.038 | .164 |
| One chronic disease | Perceived stress | Financial strain | 1.661 | 0.168 | 0.296 | < .001 |
| One chronic disease | HRQoL | Age | 0.000 | 0.000 | 0.025 | .612 |
| One chronic disease | HRQoL | Male (vs. female) | -0.014 | 0.008 | -0.052 | .076 |
| One chronic disease | HRQoL | Medium education (vs. basic) | -0.012 | 0.018 | -0.044 | .517 |
| One chronic disease | HRQoL | High education (vs. basic) | -0.018 | 0.018 | -0.067 | .319 |
| One chronic disease | HRQoL | Retired (vs. employed) | -0.021 | 0.013 | -0.065 | .105 |
| One chronic disease | HRQoL | Other non-employed (vs. employed) | -0.010 | 0.014 | -0.027 | .459 |
| One chronic disease | HRQoL | Financial strain | -0.015 | 0.005 | -0.128 | .003 |
| Multiple chronic diseases | Perceived stress | Age | -0.146 | 0.014 | -0.338 | < .001 |
| Multiple chronic diseases | Perceived stress | Male (vs. female) | -1.347 | 0.317 | -0.092 | < .001 |
| Multiple chronic diseases | Perceived stress | Medium education (vs. basic) | -0.423 | 0.508 | -0.029 | .405 |
| Multiple chronic diseases | Perceived stress | High education (vs. basic) | -0.548 | 0.567 | -0.036 | .334 |
| Multiple chronic diseases | Perceived stress | Retired (vs. employed) | 0.447 | 0.436 | 0.031 | .304 |
| Multiple chronic diseases | Perceived stress | Other non-employed (vs. employed) | 0.991 | 0.461 | 0.046 | .032 |
| Multiple chronic diseases | Perceived stress | Financial strain | 1.837 | 0.154 | 0.328 | < .001 |
| Multiple chronic diseases | HRQoL | Age | 0.001 | 0.000 | 0.067 | .066 |
| Multiple chronic diseases | HRQoL | Male (vs. female) | -0.002 | 0.008 | -0.006 | .790 |
| Multiple chronic diseases | HRQoL | Medium education (vs. basic) | 0.005 | 0.014 | 0.015 | .691 |
| Multiple chronic diseases | HRQoL | High education (vs. basic) | 0.023 | 0.014 | 0.062 | .091 |
| Multiple chronic diseases | HRQoL | Retired (vs. employed) | -0.059 | 0.010 | -0.169 | < .001 |
| Multiple chronic diseases | HRQoL | Other non-employed (vs. employed) | -0.033 | 0.011 | -0.064 | .004 |
| Multiple chronic diseases | HRQoL | Financial strain | -0.014 | 0.004 | -0.104 | < .001 |

***Note.*** Covariate estimates are shown to document the complete multigroup model. Education was dummy-coded with basic education as the reference category; employment status was dummy-coded with employed as the reference category; gender was coded 0 = female and 1 = male. CI omitted for brevity.

**Supplementary Table S5. Panel E. EQ-5D-5L ceiling distribution by chronic disease burden**

| **Indicator** | **No chronic disease** | **One chronic disease** | **Multiple chronic diseases** |
| --- | --- | --- | --- |
| EQ-5D-5L index at upper bound, weighted % | 46.1 | 23.7 | 8.4 |

***Note.*** Percentages are weighted estimates from the complete-case analytic sample. The EQ-5D-5L upper bound was defined as an index value of 1.00. Overall, 22.1% of the weighted analytic sample was at the upper bound.

**Supplementary Table S6. Sensitivity analysis using EQ-VAS as an alternative global health outcome**

| Association | B | SE | 95% CI | β | p |
| --- | --- | --- | --- | --- | --- |
| HL -> perceived stress | -0.338 | 0.019 | [-0.374, -0.302] | -0.286 | < .001 |
| Perceived stress -> EQ-VAS | -1.274 | 0.058 | [-1.387, -1.160] | -0.455 | < .001 |
| Direct association: HL -> EQ-VAS | 0.249 | 0.065 | [0.122, 0.377] | 0.075 | < .001 |
| Indirect association: HL -> perceived stress -> EQ-VAS | 0.431 | 0.031 | [0.369, 0.492] | 0.130 | < .001 |
| Total association: HL -> EQ-VAS | 0.680 | 0.064 | [0.555, 0.806] | 0.205 | < .001 |

***Note.*** N = 3,716. The same analytic sample, covariates, post-stratification weights, and estimator as in the main path model were used. EQ-VAS ranges from 0 to 100, with higher scores indicating better self-rated health. The indirect association represented 63.3% (95% CI, 50.2% to 76.4%) of the total association based on unstandardized coefficients. The corresponding proportion in the main EQ-5D-5L index model was 82.4%. R² = .348 for perceived stress and .296 for EQ-VAS
